# Retraction: Identification of RegIV as a Novel GLI1 Target Gene in Human Pancreatic Cancer

**DOI:** 10.1371/journal.pone.0298197

**Published:** 2024-01-30

**Authors:** The PLOS ONE Editors

Following the publication of this article [1, 2], concerns were raised regarding Figure 8, and additional issues were noted upon editorial follow up. Specifically,

- In Figure 8, the areas at the top of lanes 4 and 6 appear similar. The underlying image provided to the *PLOS ONE* Editors did not resolve the concerns, and its editorial assessment raised additional concerns.
- Primers for the measurement of RegIV mRNA by qPCR in human cell lines appear to target the mouse Reg4 gene according to analysis by blastn [3].
- The Results subsection titled “RegIV expression changed with GLI1 expression in PANC-1 and BxPC-3” describes qRT-PCR experimental results, referring to Figure 5; qRT-PCR experiments are also described in the Figure 5 caption. However, Figure 5 reports only protein expression data.
- Editorial assessment of data tables underlying western blot quantification in Figure 5 raised concerns regarding the accuracy of the data, and the appearance of inconsistencies between the representative images in Fig 5C and F and density values.

The first author stated that all the primary data underlying this article remain available, and they provided underlying data for Figures 4, 5, 8, and S4 for editorial assessment. The first author indicated that poor image quality may have contributed to the similarities between areas within Figure 8.

The first author acknowledged that the primer sequences included in the Materials and Methods in [1] for RegIV were incorrect, and stated that the correct primers were Forward CTGCTCCTATTGCTGAGCTG, Reverse GGACTTGTGGTAAAACCATCCAG.

The first author indicated that values in the data tables provided for western blot quantification in Figure 5 are accurate, and responded to the issues outlined above. The editors remain concerned regarding the reliability of these data.

In light of the concerns affecting multiple results that question the reliability and integrity of these data, the *PLOS ONE* Editors retract this article.

FW did not agree with the retraction and stands by the article’s findings. LX, CG, AK, GH, XX, WM, LY, YH, SH, and XW either did not respond directly or could not be reached.
